# Significance of nucleic acid testing in diagnosis and treatment of post-neurosurgical meningitis caused by multidrug-resistant *Acinetobacter baumannii*: a case report

**DOI:** 10.1186/s13256-016-1104-3

**Published:** 2016-11-03

**Authors:** Ngo Tat Trung, Trinh Van Son, Dao Thanh Quyen, Dang Thi Viet Anh, Vu Viet Sang, Nguyen Xuan Lam, Nguyen Dang Manh, Vuong Phuc Duong, Bui Tri Cuong, Quyen Dang Tuyen, Nguyen Xuan Chinh, Phan Quoc Hoan, Hoang Van Tong, Christian G. Meyer, Le Huu Song

**Affiliations:** 1 Department of Molecular Biology, 108 Military Central Hospital, Hanoi, Vietnam; 2 Institute of Clinical Infectious Diseases, 108 Military Central Hospital, No 1, Tran Hung Dao Street, Hai Ba Trung Dist, Hanoi, Vietnam; 3 Institute of Tropical Medicine, University of Tübingen, Tübingen, Germany; 4 Vietnamese-German Centre for Medical Research, 108 Military Hospital, Hanoi, Vietnam

**Keywords:** Neurosurgery, Meningitis, *Acinetobacter baumannii*, Nucleic acid testing, Bacterial culture, Hospital infection

## Abstract

**Background:**

Neurosurgery may pose the risk of patients’ developing nosocomial meningitis caused by infection with hospital pathogens. Rapid detection of the causative pathogens is essential for selecting the appropriate antibiotic treatment. However, the classical culture-based detection of bacterial infection is time-consuming and often fails to establish the correct diagnosis. Molecular techniques offer improved diagnostic means to guide the proper antibiotic therapy.

**Case presentation:**

A 32-year-old Vietnamese man underwent neurosurgery and subsequently developed meningitis. The classical bacterial culture method failed to detect any infectious agents, whereas polymerase chain reaction-based assays identified *Acinetobacter baumannii* as the causative pathogen. In addition, detection of the acquired extended-spectrum beta-lactamase gene *VEB* and carbapenem resistance genes *NDM-1* and *IMP* suggested that the isolated *A. baumannii* strain was multidrug resistant. Upon the establishment of the correct diagnosis, an adequate treatment regimen was chosen and he recovered completely.

**Conclusions:**

This case report demonstrates the usefulness of the molecular approach as an important addendum and alternative to culture-based diagnosis in order to detect the pathogen causative for meningitis, including the indicators for resistance.

## Background

Patients subjected to neurosurgery are at a high risk of developing nosocomial meningitis with lethal consequences, especially in the case of cerebrospinal fluid (CSF) leakage. Nosocomial infections with *Pseudomonas aeruginosa, Staphylococcus aureus*, and coagulase-negative staphylococci are known to be common pathogens of nosocomial meningitis in craniotomized patients [1–3]. However, application of antibiotics has induced changes in the epidemiological spectrum of the causative pathogens of meningitis following neurosurgery [4–6].

*Acinetobacter baumannii* is a Gram-negative coccobacillus that historically has been recognized as a pathogen of hot and humid climates, where it has contributed to infections in intensive care units (ICUs), and of community-acquired pneumonia [2]. *A. baumannii* can colonize skin, wounds, and respiratory and gastrointestinal tracts [7]. Recently, some reports have described the detection of multidrug resistant clones as important hospital pathogens causing serious nosocomial infections, including meningitis [8–10].

Culture systems are widely used to establish the diagnosis of bacterial infections. However, classical blood culture has several drawbacks: (i) it detects only culturable microbes, thus reducing the possibility of successfully identifying microorganisms from patients previously treated with antibiotics; (ii) it takes 24 to 48 hours to obtain first results of cultures, which hampers accurate treatment. In such situations, nucleic acid testing (NAT) methods can use bacterial genetic materials, thus providing a diagnostic alternative [11]. Here, we report on the diagnosis and treatment of a post-neurosurgical meningitis case, where classical blood culture was unable to detect the causative pathogen, whereas an in-house polymerase chain reaction (PCR) assay successfully identified *A. baumannii* as the responsible agent. In addition, the genes conferring multidrug-resistance were also recognized. As a consequence, appropriate antibiotic treatment was applied and the patient recovered completely.

## Case presentation

A 32-year-old Vietnamese man weighing 55 kg presented to our Department of Neurosurgery, 108 Military Central Hospital, Hanoi, Vietnam, in 2015 with CSF rhinorrhea as sequelae of traumatic brain injury after a traffic accident 3 years earlier. On admission, a computed tomography (CT) scan of his skull was indicated to verify any fractures and brain injury at the right frontal lobe and left occipital lobe. He underwent a craniectomy to cover a shunt identified with muscular fascia. In addition, postoperative antibiotic chemoprophylaxis was applied with 3 g cefotaxime plus 160 mg tobramycin per day (Table 1).

**Table 1:** Laboratory results of the patient and antibiotic scheme

Hospitalization time	Day 35	Day 38	Day 42	Day 45	Day 49	Day 52	Day 56	Day 59	Day 65	Day 68	Day 72
WBC (×10^6^ cell/ml)	25.04	17.5	17.9	18.1	14.5	18.35	11.4	10.0	10.5	6.6	8.56
Neutrophil (%)	94.9	80.9	79.0	80.9	75.9	86.9	79.4	77.9	63.3	69.5	78.7
Procalcitonin (ng/mL)	2.25	0.75	6.95	0.89	0.10	0.1	0.10	0.05	0.06	0.11	0.05
CSF leukocyte (cell/μL)	1800	270	2540	432	70	1395	18	47	57	31	4
CSF neutrophil (%)	95	71	89	24	20	85	71	78	2	22	NA
CSF lymphocyte (%)	5	29	NA	76	80	15	29	22	98	78	NA
CSF protein (g/L)	7	1.99	2.34	2.58	0.94	1.96	1.23	0.74	2.7	0.8	0.67
CSF glucose (mmol/L)	0.1	0.1	0.1	2.0	1.7	1.1	1.6	2.3	1.7	3.4	3
CSF culture	Neg	Neg	Neg	Neg	Neg	Neg	Neg	Neg	Neg	Neg	Neg
Urea (mmol/L)	5.0	7.1	7.0	5.5	5.4	NA	2.9	6.3	5.6	5.3	7.0
Creatinine (μmol/L)	87	58	55	51	61	NA	58	40	70	49	60
CSF PCR	Not indicated	Not indicated	Not indicated	Ab	Ab	Ab	Ab	Neg	Neg	Neg	Neg
Antibiotic therapy	Mer 1 g/6 hours + Van 1 g/12 hours			Mer 1 g/6 hours + Col 1 MIU/8 hours				Mer 2 g/8 hours + Col 2 MIU/8 hours			

*Ab Acinetobacter baumannii*, *Col* colistin, *CSF* cerebrospinal spinal fluid, *Mer* meropenem, *MIU* million international unit, *NA* not available, *Neg* negative, *PCR* polymerase chain reaction, *Van* vancomycin, *WBC* white blood cell

One week after surgery, he had fever, but no chills, headache, vomiting, stiffness of the neck or localized paralysis. The Kernig’s sign of meningitis was absent. Treatment with antibiotics was indicated with 3 g ceftizoxime plus 400 mg ciprofloxacin per day. However, fever (38 °C) persisted without further symptoms of meningitis; CSF was still leaking though his nose. The CSF was clear, with a white blood cell (WBC) count of 21,000 cells/ml, glucose concentration of 3.1 mmol/l, protein 620 mg/dl, and serum procalcitonin of 0.2 ng/ml. At that time, an external lumbar drainage was inserted to release his CSF and antibiotic treatment was changed to 3 g ceftriaxone plus 0.5 g levofloxacin daily. After 9 days at day 28 of hospitalization, he had no fever, no headache, and CSF rhinorrhea discontinued.

His normal condition lasted for 1 week only. At day 35 of his hospitalization he developed meningitis. A physical examination showed fever of 39 °C, chills, headache, vomiting, neck stiffness, impaired consciousness, and a positive Kernig’s sign. A laboratory examination revealed a WBC count of 25×10^6^ cells/ml with 95 % of neutrophils, serum procalcitonin of 2.25 ng/ml, and CSF findings of 1800 leukocytes/μl, a protein level of 7 g/ml, and a glucose level of 0.1 mmol/l. A CSF culture did not yield any microorganism (Table 1) and he was treated with 4 g meropenem plus 2 g vancomycin per day despite unconfirmed microbiological diagnosis.

**Fig. 1:**
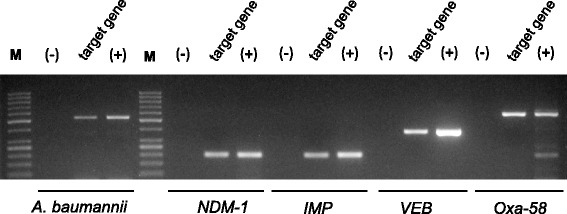
Molecular diagnostics of patient’s cerebrospinal spinal fluid. Polymerase chain reaction assays show specific amplified band for *Acinetobacter baumannii* (530 base pairs), *NDM-1* gene (200 base pairs), *IMP* gene (204 base pairs), *VEB* gene (391) and *OXA-58* gene (599). *M* marker 50 base pairs, *(−)* negative control, *(+)* positive control

One week later at day 42 of his hospitalization he still had a severe meningitis syndrome with chills, headache, vomiting, neck stiffness, reduced consciousness, positive Kernig’s sign, fever peaking at 39 °C, a WBC count of 18×10^6^/mL with 85 % neutrophils, procalcitonin of 6.95 ng/mL, and CSF containing 2540 leukocytes/μl (Table 1). The CSF culture was still negative for any microorganism. Therefore, in-house molecular assays were indicated [12], which led to the identification of genetic fragments of *A. baumannii*. Furthermore, the beta-lactam carbapenem resistance genes Vietnamese extended-spectrum beta-lactamase (*VEB*), *OXA-58,* New Delhi metallo-beta-lactamase-1 (*NDM-1*), and Imipenemase (*IMP*), respectively, were detected (Fig. 1). Thus, his diagnosis was established as meningitis due to infection with multidrug-resistant *A. baumannii*.

Following initiation of antibiotic treatment at day 45 of hospitalization with 4 g meropenem plus colistin 3 MIU per day as indicated, his clinical condition improved considerably. Fever and headache discontinued, Kernig’s sign was negative, CSF rhinorrhea halted, and the laboratory parameters (measured at day 52 of hospitalization) were WBC count 13×10^6^/mL, procalcitonin 0.1 ng/mL, CSF with 85 leukocytes/ml, and protein level 1.96 g/L. However, after 12 days of colistin-based therapy, he relapsed with typical symptoms of meningitis, including renewed fever peaks of 38 °C, headache, neck stiffness, impaired consciousness, and a positive Kernig’s sign; PCR assays of CSF samples were again positive for *A. baumannii*. Most likely, 4 g meropenem plus colistin 3 MIU per day were not effective enough to eliminate the pathogens. We decided to increase the dosage to 6 g meropenem plus colistin 6 MIU per day, which was maintained for 2 weeks. Following this increase, he recovered, and laboratory parameters were normal. A CSF PCR assay was negative for *A. baumannii*. The antibiotic treatment was continued for 3 weeks. At the last 3 weeks of hospitalization, antibiotics were no longer indicated and he did not develop any clinical signs and symptoms. He was discharged but underwent control examination every 2 weeks for a period of 3 months without a diagnosis of relapse.

## Discussion

During the past 3 decades, *A. baumannii* has emerged from being an organism of low virulence to an important infectious pathogen in hospitals worldwide. Due to its successful adaptation to warm and humid climates, *Acinetobacter* infections are clinically prevalent in tropical regions, including Vietnam. The abuse of antibiotics has resulted in an epidemiological change in spectrum of pathogenic causatives of surgical infections, including post-neurosurgical meningitis [4–6]. This may have led to some degree of multidrug resistance, as pathogens are able to accumulate diverse mechanisms of resistance to almost all antibiotics [7]. So far, *A. baumannii* appears to be an extremely rare cause of community-acquired meningitis, but it is certainly an increasingly important pathogen associated with post-neurosurgical meningitis. *Acinetobacter* species contribute to only 0·2 % of community-acquired bacterial meningitis and to about 3.6 % of hospital-acquired bacterial meningitis [8]. The mortality rate of patients with post-neurosurgical meningitis caused by carbapenem-resistant *A. baumannii* is 71 % [8]. The combination of carbapenem and colistin is the last option of choice. However, since the polymyxin-derived drug diffuses poorly only to the central nervous system and has a high nephrotoxicity and/or neurotoxicity, it is a challenge to treat patients by intravenously applying these drugs.

Although bacterial culture is widely accepted as the gold standard to detect pathogens, it is time-consuming and often fails to establish the correct diagnosis, especially in patients with blood stream infection or bacterial meningitis. In this report, although the patient showed typical symptoms of meningitis, previous treatment with several prophylactic antibiotics during his neurosurgery might have prevented positive results of the CSF culture. Thus, we were unable to initiate an optimized antibiotic regimen. However, by using our in-house molecular assays [12], we could detect genetic material of *A. baumannii*. Moreover, the associated extended spectrum beta-lactamase (*ESBL*) encoding gene (*VEB*) and carbapenemase-encoding gene *NDM-1*, and the metallo-beta-lactamase *IMP* [13] were also identified, suggesting multidrug resistance of *A. baumannii*. Based on the molecular diagnosis, the appropriate antibiotic regimen of colistin and carbapenem was indicated. However, due to the poor permeability of colistin across the central nervous system, our patient only partially responded to the treatment and remained with typical signs of meningitis. As his serum levels of urea and creatinine were in normal ranges (Table 1), indicating absence of nephrotoxicity, we decided to increase the antibiotic dosage to 6 g meropenem plus colistin 6 MIU, which eventually led to his complete recovery.

## Conclusions

This case report demonstrates the usefulness of our in-house multiplex PCR assays as an alternative way to detect pathogens causative of meningitis and the acquired antibiotic resistance genes in culture-negative situations.

## Abbreviations

CSF: Cerebrospinal fluid
ESBL: Extended-spectrum beta-lactamase
*IMP*: Imipenemase
*NDM-1*: New Delhi metallo-beta-lactamase-1
PCR: Polymerase chain reaction
*VEB*: Vietnamese extended-spectrum beta-lactamase
WBC: White blood cell
